# Peripartum Hysterectomy among Patients Admitted to the Department of Obstetrics and Gynaecology in a Tertiary Care Centre: A Descriptive Cross-sectional Study

**DOI:** 10.31729/jnma.8162

**Published:** 2023-05-31

**Authors:** Sushma Lama, Sushila Todi, Reena Shrestha, Swikrity Acharya

**Affiliations:** 1 Department of Obstetrics and Gynaecology, Patan Academy of Health Sciences, Lagankhel, Kathmandu, Nepal

**Keywords:** *caesarean section*, *hysterectomy*, *placenta accreta*

## Abstract

**Introduction:**

Emergency peripartum hysterectomy is a life-saving procedure performed as an emergency procedure to control torrential bleeding and it is associated with significant maternal morbidity and mortality. There are only a few studies regarding this topic so this study guides us to monitor the trend and start appropriate policies to reduce unnecessary caesarean deliveries. The aim of this study was to find out the prevalence of peripartum hysterectomy among patients admitted to the Department of Obstetrics and Gynaecology in a tertiary care centre.

**Methods:**

A descriptive cross-sectional study was conducted in the Department of Obstetrics and Gynaecology of the tertiary care centre. Data from 1 January 2015 to 31 December 2022 were collected between 25 January 2023 and 28 February 2023 from the hospital records. Ethical approval was obtained from the Institutional Review Committee of the same institute (Reference number: 2301241700). Convenience sampling was done. Point estimate and 95% Confidence Interval were calculated.

**Results:**

Among 54,045 deliveries, peripartum hysterectomy was seen in 40 (0.074%) (0.05-0.10, 95% Confidence Interval). The major indication of emergency peripartum hysterectomy was abnormal placentation (placenta accreta spectrum) which was 25 (62.50%) followed by uterine atony in 13 (32.50%) of patients and uterine rupture in 2 (5%).

**Conclusions:**

The prevalence of peripartum hysterectomy was lower than in other studies done in similar settings. The indication for emergency peripartum hysterectomy has changed in recent years from uterine atonicity to the morbidly adherent placenta which is due to a rise in the caesarean section rate.

## INTRODUCTION

Peripartum hysterectomy is a life-saving emergency obstetric procedure.^1^ It is performed after vaginal delivery or at the time of caesarean birth where conservative measures do not control haemorrhage like fundal massaging, bimanual uterine compression, use of uterotonics, uterine packing, compression sutures and ligation of the uterine artery.^2^ Incidence of peripartum hysterectomy around the world is 0.64 to 5.09 per 1,000 deliveries.^3^

Earlier the most common indication for hysterectomy was uterine atony but these days it is due to abnormal placentation.^4,5^ This rise has been seen in parallel to rising cesarean section (CS) which increases the risk of peripartum hysterectomy.^6,7^ In Nepal, there are only a few studies regarding this topic so this study guides us to monitor the trend and apply policies to reduce unnecessary CS.

The aim of this study was to find out the prevalence of peripartum hysterectomy among patients admitted to the Department of Obstetrics and Gynaecology in a tertiary care centre.

## METHODS

This descriptive cross-sectional study was done in the Department of Obstetrics and Gynaecology of Patan Academy Of Health Sciences (PAHS), Lagankhel, Kathmandu, Nepal. Data from 1 January 2015 to 31 December 2022 were collected between 25 January 2023 and 28 February 2023 from the hospital records. Ethical approval was obtained from the Institutional Review Committee of the same institute (Reference number: 2301241700). All pregnant women admitted to the maternity ward within the study period were included in the study. Women who delivered before 28 weeks and underwent a hysterectomy, or peripartum hysterectomy done outside Patan Hospital and referred to the intensive care unit were excluded from the study. Convenience sampling was done. The sample size was calculated by using the following formula:


$$\begin{array}{ll} \text{n} & ={\text{Z}}^{2}\times \frac{\text{p}\times \text{q}}{{\text{e}}^{\text{2}}}\\ & =1.96^{2}\times \frac{0.50\times 0.50}{0.01^{2}}\\ & =9604 \end{array}$$

Where,

n = minimum required sample size
Z = 1.96 at 95% Confidence interval (CI)
p = prevalence taken as 50% for maximum sample size calculation
q = 1-p
e = margin of error, 1%

The minimum sample size obtained was 9604. The sample size was quintupled which was 48,020. However, 54,045 patients were included in this study.

Data were collected from the patient's file regarding maternal age, parity, gestational age, type of delivery, an indication of hysterectomy, risk factors, and maternal complications were noted. Data were entered and analysed by using Microsoft Excel 2016. Point estimate and 95% CI were calculated.

## RESULTS

Among 54,045 deliveries, the prevalence of peripartum hysterectomy was 40 (0.074%) (0.05-0.10, 95% CI). The mean age was 30.5±4.15 years. The majority of the patients were multigravida 32 (80%). The mean gestational age was 35.60±3.12 weeks (Table 1).

**Table 1:** Distribution of age, parity and gestational age (n= 40).

Parameters	n (%)
**Age (years)**	
20-25	5 (12.50)
26-29	9 (22.50)
30-35	20 (50)
>35	6 (15)
**Gravida**	
Primigravida	8 (20)
Multigravida	32 (80)
**Gestational age (Weeks)**	
28-33	6 (15)
34-36	13 (32.50)
≥37	21 (52.50)

Total 8 (20%) peripartum hysterectomy were performed after vaginal delivery and 32 (80%) were performed after CS. All patients 40 (100%) received oxytocin, ergotamine derivative, and condom tamponade were inserted in 2 (5%) patients, uterovaginal packing in 2 (5%) patients and vaginal tamponade in 1 (2.50%) patient. There were 5 (12.50%) EPH following induction of labour. The major indication of EPH was abnormal placentation (placenta accreta spectrum) which accounted for 25 (62.50%) cases among which, 1 (2.50%) led to massive blood loss, shock and disseminated intravascular coagulation (Table 2).

**Table 2:** Distribution of maternal outcome (n= 40).

Parameters	n (%)
**Mode of delivery**	
Vaginal	8 (20)
Caesarean section	32 (80)
**Indication of hysterectomy**	
Rupture uterus	2 (5)
Atonicity	13 (32.50)
Placenta accreta spectrum	25 (62.50)

Postpartum haemorrhage was seen in 39 (97.50%) cases. The minimum blood loss was 400 ml and the maximum was up to 4000 L. The patients who had 400 ml of blood were due to bilateral internal iliac balloon tamponade insertion by an interventional radiologist. Almost all 39 (97.50%) patients received a massive blood transfusion. There were 5 (12.50%) patients with bladder injury. About 1 (2.50%) patient had renal failure for which 12 cycles of hemodialysis were done. A total of 3 (7.50%) patients needed relaparotomy due to hemoperitoneum caused by vaginal cuff bleeding (Table 3).

**Table 3:** Maternal complications (n= 40).

Complications	n (%)
**Postpartum haemorrhage (ml)**	40 (100)
<750	1 (2.50)
750-1500	7 (17.50)
1600-200	19 ( 47.50)
>2000	13 (32.50)
Disseminated intravascular coagulation	6 (15)
Bladder injury	5 (12.50)
Relaparotomy	3 (7.50)
Wound infection	3 (7.50)
Mortality	3 (7.50)
Renal failure	1 (2.50)

The most common underlying condition of EPH was placenta previa 25 (62.50%) (Table 4).

**Table 4:** Underlying conditions (n= 40).

Parameters	n (%)
Placenta previa	25 (62.50)
Previous 1 CS	16 (40)
Previous 2 CS	4 (10)
Dilatation and curettage	1 (2.50)
Instrumental delivery	1 (2.50)

## DISCUSSION

Peripartum hysterectomy causes significant maternal morbidity and mortality and it is the most challenging procedure that an obstetrician can face. A timely decision to perform a peripartum hysterectomy is very important as delay can lead to further haemorrhage and anaemia which is responsible for high maternal mortality. The prevalence of peripartum hysterectomy differs worldwide. In Nigeria^8^ it is (0.51%), in China^9^ (0.22%), and in another study from India^10^ it is (0.52%). Out of 54,045 deliveries, there were 40 peripartum hysterectomies hence the prevalence was 0.074% in our study. A similar study showed an incidence of 1.48 per thousand which was higher than our study.^11^

Our study showed the main indication of EPH was placenta accreta spectrum 62.5% followed by uterine atony 32.5% but another study showed rupture uterus was the common indication.^11^ Several studies have shown that abnormal placentation is the major cause of EPH, replacing uterine atony.^4,5^ This is due to the successful management of uterine atony with uterotonics, embolization, B-lynch suture, condom tamponade and selective devascularisation. In this study, all women with a history of the previous caesarean section that underwent EPH also had abnormal placentation. Similar study found that women with previous CS had an 8-fold risk of abnormal placentation in the next pregnancies.^12^

In a study done in UK almost one-fifth of cases (19.6%) underwent relaparotomy whereas in our study there were 3 (7.5%) relaparotomy for hemoperitoneum.^13^

A similar study showed in patients who underwent EPH, 80.6% were delivered by caesarean section and 19.4% were delivered vaginally.^14^ This clearly reflects the association between having a previous CS and abnormal placentation in consequent pregnancies. In our study, 8 peripartum hysterectomies were performed after vaginal delivery and 32 were performed after caesarean section suggesting that caesarean delivery has a significant association with peripartum hysterectomy.

There were 3 (7.50%) mortality in our study which occurred immediately during the postoperative period. In 5% patients it was due to atonicity and in 2.50% due to placenta accreta spectrum which led to massive blood loss, shock and disseminated intravascular coagulation. Another study showed three mortality due to haemorrhagic shock.^11^ In another study showed there was no mortality death report.^14^

Incidence of urinary tract injury in studies from the UK,^13^ China,^9^ and another centre from India^10^ were 12.2%, 4.1%, and 7.93% respectively. In our study bladder injury was among the most commonest complication which was 12.5% which occurs due to dense adhesion of the bladder to the lower uterine segment or when associated with placenta accreta spectrum distorting the normal anatomy.

In our study we had done bilateral internal artery balloon tamponade in one patient with the help of an interventional radiologist before the surgery in which there was blood loss of only 400 ml and blood transfusion was not required. A similar study showed that internal iliac artery balloon tamponade decreases blood transfusion requirements among women requiring emergency EPH for placenta accreta. There was a significant reduction in the total number of blood products transfused (3.5 units vs. 15 units). Further studies are required to clarify the potential benefits of balloon tamponade in the elective setting.^15^

There are some limitations to our study. Since EPH is rarely performed the number of patients in our study was relatively small. A multicenter-based larger population study might have a different outcome compared to that of this study. We did internal iliac artery balloon tamponade only in one patient if this procedure could be routinely done in all patients with the help of the interventional radiologist team the result would have been different and there would be less morbidity and mortality.

## CONCLUSIONS

The prevalence of peripartum hysterectomy was lower than in other studies done in similar settings. The risk factors of peripartum hysterectomy should be identified antenatally and the high-risk group should be delivered in a tertiary care centre with a blood bank and ICU facility, trained experienced surgeon, anaesthesiologist, and medical team. Based on our study, we are concerned that the rate of EPH will continue to rise in the future if actions are not taken to prevent unnecessary caesarean deliveries. Therefore, protocol and guidelines have to be made at the national level which will guide the clinician to monitor the trend and start applying appropriate policies in order to reduce caesarean deliveries.
